# Characterization of Gastric and Neuronal Histaminergic Populations Using a Transgenic Mouse Model

**DOI:** 10.1371/journal.pone.0060276

**Published:** 2013-03-29

**Authors:** Angela K. Walker, Won-Mee Park, Jen-Chieh Chuang, Mario Perello, Ichiro Sakata, Sherri Osborne-Lawrence, Jeffrey M. Zigman

**Affiliations:** The Department of Internal Medicine (Division of Hypothalamic Research and Division of Endocrinology & Metabolism) and the Department of Psychiatry, The University of Texas Southwestern Medical Center, Dallas, Texas, United States of America; University of Cordoba, Spain

## Abstract

Histamine is a potent biogenic amine that mediates numerous physiological processes throughout the body, including digestion, sleep, and immunity. It is synthesized by gastric enterochromaffin-like cells, a specific set of hypothalamic neurons, as well as a subset of white blood cells, including mast cells. Much remains to be learned about these varied histamine-producing cell populations. Here, we report the validation of a transgenic mouse line in which Cre recombinase expression has been targeted to cells expressing histidine decarboxylase (HDC), which catalyzes the rate-limiting step in the synthesis of histamine. This was achieved by crossing the HDC-Cre mouse line with Rosa26-tdTomato reporter mice, thus resulting in the expression of the fluorescent Tomato (Tmt) signal in cells containing Cre recombinase activity. As expected, the Tmt signal co-localized with HDC-immunoreactivity within the gastric mucosa and gastric submucosa and also within the tuberomamillary nucleus of the brain. HDC expression within Tmt-positive gastric cells was further confirmed by quantitative PCR analysis of mRNA isolated from highly purified populations of Tmt-positive cells obtained by fluorescent activated cell sorting (FACS). HDC expression within these FACS-separated cells was found to coincide with other markers of both ECL cells and mast cells. Gastrin expression was co-localized with HDC expression in a subset of histaminergic gastric mucosal cells. We suggest that these transgenic mice will facilitate future studies aimed at investigating the function of histamine-producing cells.

## Introduction

Whether assisting with the digestive processes of the stomach, signaling as a neurotransmitter in the central nervous system, or inducing an inflammatory reaction, the biogenic amine histamine plays a major role in numerous behavioral and physiological processes [1], [2]. In order to generate histamine, cells utilize the enzymatic activity of histidine decarboxylase (HDC), which acts to decarboxylate the amino acid L-histidine to form histamine [3], [4], [5]. Since HDC is essential for the proper regulation of histamine formation and release, the ability to identify cells that contain HDC is central to investigating the physiology of the histaminergic systems of the body.

The gastric mucosa of the stomach contains a highly regulated population of histaminergic cells known as enterochromaffin-like (ECL) cells [6]. Classically, ECL cells have been distinguishable from other gastric mucosal cell populations by the distinctive electron microscopic appearance of their secretory granules [7], [8]. In rodents, ECL cells store approximately 80% of the total gastric histamine content, and thus, account for the majority of histaminergic cells in the oxyntic mucosa [9]. One of the best characterized actions of ECL cell-derived histamine is the stimulation of gastric acid secretion from nearby parietal cells [6], [10], which not only plays a pivotal physiologic role in digestion, but also plays a key pathologic role in the occurrence of dyspepsia, gastroesophageal reflux disease, and peptic ulcer disease [11], [12], [13], [14], [15]. Blockade of H_2_-type histamine receptors has long been utilized as one of the major means of treating those ailments and emphasizes the importance of ECL cell-derived histamine [14], [16]. Among the important mechanisms found to regulate ECL function, endocrine, paracrine, and neural pathways all help direct ECL cell histamine release [17]. Of importance, gastrin stimulation of calcium signaling largely mediates histamine release, and pituitary adenylate cyclase activating peptide (PACAP) regulates ECL cell function through activation of PACAP type 1 receptors (PAC1) [17], [18], [19]. Despite a vast literature on ECL cells, the sparse and dispersed nature of their distribution within the stomach as well as the lack of an identifier more practical than the appearance of their secretory granules under electron microscopy has made study of this cell type challenging.

Mast cells comprise another important population of histamine-producing cells within the stomach [20]. Derived from the bone marrow, mast cells are found not only in the stomach but also in the many connective tissues throughout the body, as well as the brain and dura [2], [21]. In the mast cell immune response, immunoglobulin E (IgE) attached to mast cells binds to antigens, thus resulting in degranulation and release of histamine from the cells [20]. In the gastric mucosa, the release of mast cell histamine typically activates vascular H_1_ receptors to encourage extravasation and chemotaxis [20]. Mast cells in the stomach have been implicated in contributing to increased gastric acid secretion, irritable bowel syndrome, polyp formation, and tumor angiogenesis [22]. Some but not all studies suggest that gastric mast cells also contribute to the development of peptic ulcers, gastric carcinomas, and gastric injury [23], [24], [25]. In response to gastrointestinal parasites, mast cells play an important role in host defense, assisting in parasite expulsion and rejection [26], [27]. However, the importance of gastric mast cells in various physiologic as well as pathogenic stomach inflammatory responses has only begun to be explored.

Histamine also serves as an important signal within the central nervous system. Histaminergic neurons are concentrated in a small region of the posterior basal hypothalamus known as the tuberomamillary nucleus [2], [28], [29], [30]. As would be predicted from the widespread histaminergic efferent projections from the tuberomamillary nucleus, histamine receptors are found widely distributed throughout the brain [2], [28], [29]. Such suggests a heavy influence of histamine signaling on many different brain regions and on a variety of behaviors [31], [32]. Perhaps the best characterized function of central histamine is related to its effects on arousal state: activation of histaminergic neurons increases wakefulness while inhibition of these neurons has a sedating effect [2]. CNS histamine also regulates several other homeostatic processes, including thermoregulation, feeding, drinking, energy metabolism, nociception, and biological rhythms, to name a few, and also influences higher brain functions such as mood and cognition [2]. With such an extensive range of physiological and behavioral effects, the idea of histamine neurons in the tuberomamillary nucleus as distinct subpopulations rather than a single unit of cells is beginning to gain interest [33]. Of note, the histaminergic neuron system has historically been divided into five distinct cell clusters based on regional location within the tuberomamillary nucleus, termed E1–E5, and several recent studies suggest the existence of functional distinctions between these five nuclei [29], [33], [34], [35]. To elaborate, different types of stress such as restraint stress, foot shock, or hypoglycemia have been found to activate histamine neurons in the rostral subgroups (E4–E5) rather than the caudal subgroups (E1–E3), while CO_2_ exposure or acidification in rat brain slices causes excitation in the ventrolateral E2 neurons [36], [37], [38].

Although several key functions and regulatory pathways already have been established regarding histaminergic systems of the brain and stomach, much remains to be discovered. Recently, we reported the generation of a novel HDC-Cre mouse model in which Cre recombinase is expressed within histidine decarboxylase-containing cells [37]. We previously demonstrated co-expression of Cre recombinase activity within about 74% of ventrolateral (E2) HDC-immunoreactive neurons HDC [37]. However, expression of the transgene was not explored in the other four tuberomamillary nucleus subgroups or in the stomach for that initial study. The aim of the current study was to more fully validate and quantify the specific expression of Cre recombinase in the stomach and brains of our HDC-Cre mouse model. Indeed, Cre recombinase activity localized within the vast majority of HDC-immunoreactive cells populating the stomach and the various tuberomamillary nucleus subgroups. Furthermore, HDC expression within highly purified populations of gastric mucosal cells expressing the transgene, as made possible by fluorescence activated cell sorting, was found to coincide with other markers of both ECL cells and mast cells. We suggest that these transgenic mice will facilitate future studies aimed at investigating the function of histamine-producing cells.

## Materials and Methods

### Ethics Statement

This study was carried out in strict accordance with the guidelines included in the Guide and Care for Use of Laboratory Animals. All protocols were approved by the Institutional Animal Care and Use Committee of UT Southwestern Medical Center under the permit numbers 2009-0377 and 2008-0107. Methods to minimize suffering were performed such as the use of chloral hydrate for anesthetization of animals during non-survival surgeries.

### Generation and Care of HDC Reporter Mice

HDC-Cre transgenic mice were generated by microinjection of a Cre-modified, mouse HDC bacterial artificial chromosome into pronuclei of fertilized one-cell stage embryos of C57BL6/J mice. The construction of the HDC-Cre transgene is described in more detail elsewhere [37], but in brief involved replacing a portion of the HDC gene encoding the first 47 amino acids of HDC and the interventing intron 1 with the coding sequence of Cre recombinase. Such was accomplished in an HDC bacterial artificial chromosome (RP24-141N14; BACPAC Resources Center at Children's Hospital Oakland Research Institute) which spanned the entire coding region of HDC plus approximately 92.42 kb sequence upstream of the HDC start codon and approximately 13.91 kb sequence downstream of the HDC stop codon [37]. As mentioned, an initial characterization of this novel transgenic line, limited to confirmation of Cre recombinase activity in the ventrolateral tuberomamillary nucleus, was previously described [37]. For all of the studies performed in the current report, HDC-Cre transgenic mice were bred to Rosa26-lox-STOP-lox-tdTomato reporter mice [B6J/N.Cg-Gt(ROSA)26Sortm14(CAG–tdTomato); stock #007908; The Jackson Laboratory, Bar Harbor, Maine], in which a transcriptional stop cassette is removed only in the presence of Cre recombinase activity, thus resulting in the expression of tdTomato (Tmt) fluorescence. Mice with one copy of each transgene are herein designated as HDC/Tmt mice. Animals were fed standard chow diet (Teklad Global Diet 16% Protein Diet [2016]; Harlan Teklad, Madison, WI). They had free access to water and were housed under 12 hours of light/12 hours of dark per day in a temperature-controlled environment.

### Tissue Preparation for Immunohistochemistry

Tissue preparation for histological experiments was performed as described previously [39]. Briefly, for immunohistochemistry, adult (8–15 weeks old) mice were anesthetized with chloral hydrate (500 mg/kg), injected intraperitoneally, and then perfused transcardially with diethylpyrocarbonate (DEPC)-treated 0.9% saline followed by 10% neutral buffered formalin. The stomachs and brains were removed, and after being stored in formalin for 4–6 hours at 4°C, were placed into 30% sucrose-DEPC-treated phosphate buffered saline (PBS) overnight. Stomachs were then embedded in Tissue-Tek OCT compound (Sakura Finetechnical Co., Ltd, Tokyo) and sectioned transversely at a thickness of 12 µm onto SuperFrost slides (Fisher Scientific, Pittsburg, PA) using a cryostat. Five equal series of 20 µm-thick coronal brain sections were collected using a sliding microtome and stored at −20°C in cryoprotectant until use for immunohistochemistry.

### Histochemistry of Stomach Sections

#### Visualization of Tmt Fluorescence

Slides with stomach sections were coverslipped with Vectashield Hardset Mounting Media (Vector Labs, Burlingame, CA) and examined under an Olympus IX51 microscope (Center Valley, PA), and photomicrograph images were taken of endogenous Tmt fluorescence using the Olympus DP Controller program.

#### Immunohistochemistry for HDC

After air-drying at 4°C overnight, the slides with stomach sections were washed in PBS three times and pretreated with 1% sodium dodecyl sulfate (SDS)-PBS for 5 min at room temperature. The slides were incubated with a rabbit polyclonal anti-HDC antibody (1∶500; Cappel, Aurora, OH) overnight at room temperature. After washes with PBS, the slides were incubated in Alexa Fluor 488® donkey anti-rabbit IgG (Molecular Probes, Carlsbad, CA) for 1 hr at room temperature. Labeled samples were coverslipped with Vectashield HardSet Mounting Media (Vector Labs, Burlingame, CA), and examined under a Zeiss fluorescence microscope with ApoTome attachment (Zeiss Axio Imager Z1; Thornwood, NY). Photomicrograph images were taken using AxioVision software. The specificity of the immunohistochemistry reaction was tested by omitting the primary antibody; no labeling was detected in these control reactions.

#### Toluidine Blue Histochemistry

The Olympus IX51 microscope was used to visualize endogenous Tmt fluorescence within slide-mounted stomach tissue sections for prior to staining. Mast cells were identified based on their Tmt fluorescence and their characteristic oval-shaped, “fried egg” appearance, in which a dim fluorescent cytoplasm appears surrounding a more intensely fluorescent nucleus [40]. Photomicrograph images of the mast cells were taken and the locations of the cells were carefully noted in order to pinpoint the same cells after staining. Slides with sections were then incubated in a Toluidine blue solution comprised of Toluidine blue O (Sigma-Aldrich, St. Louis, MO) in 70% ethanol and 1% NaCl (pH 2.3). After 3 min, sections were washed in distilled water, and then dehydrated with 95% and 100% ethanol, followed by xylene and coverslipping. After staining, cells which were captured in the photomicrograph images taken before the toluidine blue staining protocol were again visualized under brightfield on the microscope. New photomicrograph images were taken to capture Toluidine blue staining in those cells that had previously displayed Tmt fluorescence.

#### Dual-label Immunohistochemistry for HDC and Gastrin

For double labeling, slides were pretreated with 1% sodium dodecyl sulfate (SDS)-PBS for 5 min at room temperature prior to being incubated simultaneously with a guinea pig polyclonal anti-gastrin antibody (1∶500, Acris, San Diego, CA) and the rabbit polyclonal anti-HDC antibody (1∶500). After washes with PBS, the slides were incubated in Alexa Fluor 488® donkey anti-rabbit IgG (Molecular Probes, Carlsbad, CA) together with Alexa Fluor 594® goat anti-guinea pig IgG (Molecular Probes) for 1 hr at room temperature. After coverslipping with Vectashield HardSet Mounting Media (Vector Labs, Burlingame, CA), slides were examined under a Zeiss fluorescence microscope with ApoTome attachment (Zeiss Axio Imager Z1; Thornwood, NY), and photomicrograph images were taken with the Axiovision software. The specificity of the immunohistochemistry reaction was tested by omitting the primary antibody; no labeling was detected in these control reactions.

### Stomach Mucosal Cell Isolation and Fluorescence Activated Cell Sorting (FACS)

A combined enzymatic and mechanical dispersion method was used to prepare isolated stomach mucosal cells, as previously reported [39], [41]. Briefly, mice were quickly killed with cervical dislocation and the stomachs were removed, inverted and inflated. The inflated stomachs were incubated in 2.4 U/mL Dispase II solution (Roche Diagnostic Corporation, Indianapolis, IN) for 1.5 hours. A secondary enzymatic digestion of cell suspensions with 0.25% trypsin-EDTA (Gibco, Gaithersburg, MD) for 5 min was followed by filtration through a 100 µm nylon mesh. Cells were re-suspended in FACS buffer (3% FBS, 0.5 mM EDTA, 0.1% BSA, 10 U/mL DNaseI, 20 mg/mL glucose in HBSS) and re-filtered through a 35 µm filter right before the sort. Dissociated single cells obtained from HDC/Tmt mice were separated by a FACS Aria (Becton Dickinson, San Jose, CA) at the UTSW Medical Center Flow Cytometry Multi-user Core Facility. Cells were sorted into a Tmt-enriched population and a Tmt-negative population based on size, complexity and intensity of Tmt fluorescence (at 585 nm) and fluorescence at 530 nm. Ten independent FACS preparations (4–7 mice consisting of both males and females were used for each preparation) were included in the subsequent analyses. Depending on the preparation, these Tmt-enriched and Tmt-negative pools corresponded to slightly different percentages of sorted, living cells. The Tmt-enriched pools corresponded to 0.3% to 1.1% of sorted living cells, and the Tmt-negative pools corresponded to 20% to 97% of sorted, living cells. For “mucosa” samples, non-sorted, dissociated cells from C57BL/6J mice were obtained using the same protocol minus the FACS separation.

### RNA Extraction and Quantitative PCR

The method for RNA extraction of FACS-sorted cells and quantitative PCR was similar to that reported previously [39], [42]. The Tmt-enriched and Tmt-negative pools were adjusted after each FACS separation to contain a matched number of cells ranging from 20,000 to 50,000, and then were submitted to the RNA extraction protocol below. For the whole mucosa preparations, the entire populations of non-sorted gastric mucosal cells from each of 3 separate C57BL/6J mice were independently submitted to the RNA extraction method below. Total RNA was extracted using centrifugation with STAT60 for lysing of the cells, phenol-chloroform for phase separation, and isopropanol for precipitation of the pellet. The RNA was incubated in isopropanol at 4°C overnight prior to centrifugation in order to maximize the amount of total RNA precipitated. Lastly, RNA pellets were washed with 75% ethanol in DEPC-PBS, reconstituted in RNAlater (Ambion, Naugatuck, CT), and the concentration and relative purity of the RNA was determined using a NANODROP 1000 spectrophotometer (Thermo Fisher Scientific, Waltham, MA). Total RNA was stored at −80°C until use. After DNase I (Quiagen, Valenica, CA) treatment, complementary DNA (cDNA) was synthesized from 500 ng–1 µg total RNA using Superscript III reverse transcriptase (Invitrogen). For qPCR, 25 ng cDNA was loaded per well with iTaq SYBR Supermix (Bio-Rad Laboratories, Hercules, CA) for amplification and detection; mRNA levels for genes of interest were measured with an ABI 7300 Real-Time PCR System (Applied Biosystems, Foster, City, CA). Previously designed and validated primers are as follows [39]: HDC : 5′-GCGACCCTTCCTTCGAAATT-3′; 5′-CCTTTAACACACTTTCTGTGAGACAAT-3′, tryptophan hydroxylase 1: 5′-CCTTGGAGCTTCAGAGGAGA-3′; 5′- CAGCTGTCCATCTTGTTTGC-3′, gastrin: 5′-TCCAGGGTCCTCAACACTTC-3′; 5′-CCAAAGTCCATCCATCCGTA-3′, ghrelin: 5′-GTCCTCACCACCAAGACCAT-3′; 5′-TGGCTTCTTGGATTCCTTTC-3′, chromogranin A: 5′-GCAGGCTACAAAGCGATCCA-3′; 5′-CTCTGTCTTTCCATCTCCATCCA-3′, prohormone convertase1/3: 5′-GGCACCTGGACATTGAAAATTAC-3′; 5′-TTCATGTGCTCTGGTTGAGAAGA-3′, prohormone convertase 2: 5′-CAAGCGGAACCAGCTTCA-3′; 5′-ATTCCAGGCCAACCCCA-3′, H+/K+ ATPase β-subunit: 5′-CCCAGCTTCGGCTTCGA-3′; 5′-TGGAGACTGAAGGTGCCATTG-3′, gastric intrinsic factor: 5′-GAAAAGTGGATCTGTGCTACTTGCT-3′; 5′-AGACAATAAGGCCCCAGGATG-3′, pepsinogen F: 5′-GAAGTGGCTCTGGGTCCTT-3′; 5′-GGCTTTCCCGCAGGTTTT-3′, calpain 8: 5′-TTCTGCCTGAGGGTGTTCTC-3′; 5′- TCTTCTCCATCCATGTCACG-3′, ′; 5′-CCTTTAACACACTTTCTGTGAGACAAT-3′, somatostatin: 5′-CCCAGACTCCGTCAGTTTCT-3′; 5′-GGGCATCATTCTCTGTCTGG-3′, and cyclophilin: 5′-TGGAGAGCACCAAGACAGACA -3′; 5′-TGCCGGAGTCGACAATGAT-3′.

Newly designed primers were created to span exon-exon junctions to reduce amplification of contaminating DNA, and the efficiency and specificity of these primer pairs were confirmed by generating titration slopes and dissociation curves. These primers are as follows: Tpsb2: 5′-CTTCCCCCAGGGACATC-3′; 5′-GGAGAGGCTCGTCATTATCAATG-3′, PAC1: 5′-GCAGTCATTGCTTCGTTTCCA-3′; 5′-AGAGGTATAGGCCTTCAATGAACAG-3′, and Tmt 5′-CACCATCGTGGAACAGTACGA-3′; 5′-GCCATGCCCCAGGAACA-3′.

The PCR protocol included an initial template denaturation for 3 min at 95°C. The cycle profiles were programmed as follows: 15 sec at 95°C (denaturation) and 45 sec at 60°C (annealing and extension). Forty cycles of the profile were run and cyclophilin was used for normalization.

Of note, a separate quantitative PCR analysis was performed to confirm the appropriateness of using cyclophilin as the housekeeping gene for normalization of results. Two independent FACS preparations (5–6 mice consisting of both males and females were used for each preparation) were included. As above, equal amounts of cDNA (25 ng) prepared from each of the two new sets of Tmt-enriched and Tmt-negative pools were amplified with primers for cyclophilin and two other housekeeping genes, β-actin (5′- CATCGTGGGCCGCCCTA -3′; 5′- CACCCACATAGGAGTCCTTCTG -3′) and GAPDH (5′- CAAGGTCATCCATGACAACTTTG -3′; 5′- GGCCATCCACAGTCTTCTGG -3′). The mean threshold cycle values for cyclophilin in the Tmt-enriched and Tmt-negative pools were essentially the same, without a statistically significant difference (19.80±0.26 and 19.66±0.29, respectively). Similar threshold cycles for β-actin and GAPDH were also observed in the Tmt-enriched and Tmt-negative pools (16.12±0.07 and 16.28±0.03, β-actin; 17.51±0.23 and 17.04±0.07, GAPDH).

### Immunohistochemistry of Brain Sections

Brain tissue sections were mounted onto SuperFrost slides with PBS and allowed to dry overnight prior to staining. Slides with tissue sections were rehydrated in PBS three times, pretreated with 1% SDS-PBS for 12 min, and washed three times in PBS (total 45 min). Slides were then incubated in 3% normal donkey serum (Equitech-Bio, Kerrville, TX) with 0.3% Triton X-100 in PBS for 1 hr, followed by an overnight incubation at room temperature in rabbit anti-HDC antiserum (1∶500, Cappel) in 0.3% Triton X-100-PBS at a concentration of 1∶50. After washing in PBS, slides were incubated in Alexa Fluor 488® donkey anti-rabbit IgG (1∶300; Molecular Probes, Carlsbad, CA) for 1 hr at room temperature. Following some final PBS washes, sections were mounted in Vectashield with DAPI (Vector Laboratories, Burlingame, CA) and viewed using microscopy. Tmt fluorescence was visualized using the Zeiss Axioskop 2 microscope and was quantified in sections after the stain was performed in order to determine co-localization. Photomicrograph images were taken using Axiovision software. Omission of the primary antibody as a control experiment revealed a lack of staining in these tissues.

### Analysis of Data

The method for quantification of co-localization was carried out as performed previously [39]. To determine the degree of overlap between Tmt-positive cells and HDC immunoreactivity in the stomach, four different section planes of oxyntic mucosa, each separated by ≥70 µm along the extent of the gastric corpus, were viewed in each of five mice. To determine the degree of overlap between HDC-immunoreactive cells and gastrin-immunoreactive cells, eight different section planes of the gastric antrum were viewed in each of three mice. At each section plane, analyses were done with both 20X and 40X Zeiss Plan-Apochromat objectives from at least five fields, each 480 µm×480 µm in area, over the whole circumference of the stomach. For visualization of brain tissue sections, every fifth brain section containing the tuberomamillary nucleus was analyzed in three mice, and co-localization of HDC immunoreactivity and Tmt fluorescence was determined in the histaminergic regions E1–E5. Analysis of both the stomach and brain consisted of counting the total number of Tmt-fluorescent cells, HDC-immunoreactive cells, and cells with co-localization of HDC-immunoreactivity and Tmt fluorescence. Quantification was performed manually while visualizing live through the microscope. Using these cell counts, the percentage of HDC-immunoreactive cells containing Tmt fluorescence was calculated ([# of cells with co-localization/total # of HDC-immunoreactive cells] x 100); the percentage of Tmt-fluorescent cells with HDC-immunoreactivity was also calculated ([# of cells with co-localization/total # of Tmt-fluorescent cells] x 100). Images of sections were taken using either the green filter, the red filter, or both. Adobe PhotoShop 7.0 (San Jose, CA) was used to adjust contrast, brightness, and color of the photomicrographs.

For qPCR analysis, one-way ANOVAs were performed; Dunnett's post-hoc analysis was used to compare expression levels in the sorted cell populations with those in whole mucosa. Statistics were performed using GraphPad Prism 5.0. P<0.05 was considered statistically significant, and all data are expressed as mean ± standard error of the mean.

## Results

### Stomach expression of Cre recombinase activity

**Figure 1:**
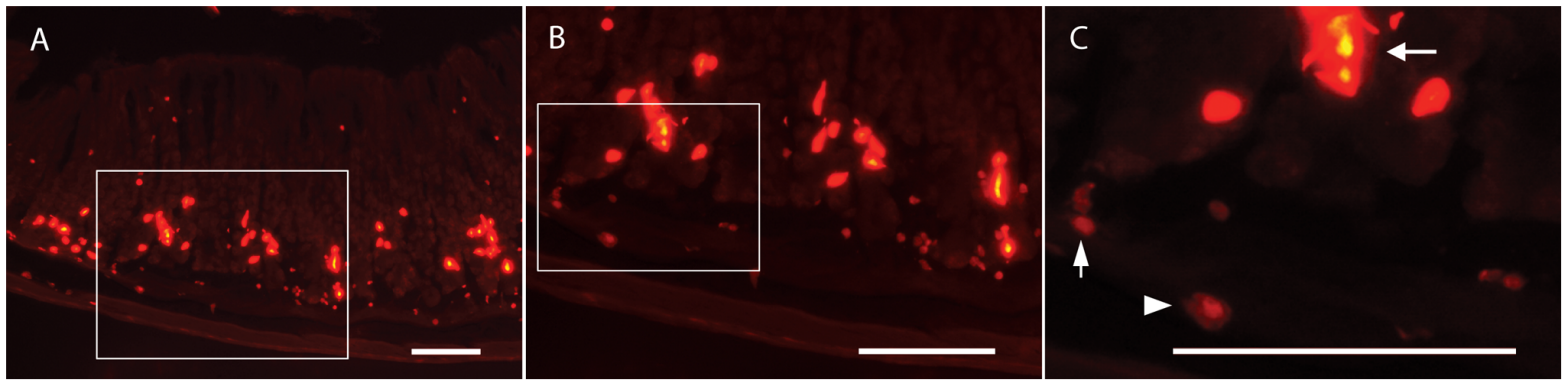
Expression of Tmt within the gastric oxyntic mucosa and submucosa of HDC/Tmt mice. A) Low magnification photomicrograph showing expression of cells with different intensities of Tmt fluorescence in the gastric mucosa, submucosa, and muscularis layers. B–C) High magnification of same section reveals distinct shapes of different cell types: leftward-facing arrow points to a representative intensely-fluorescent, elongated cell (ECL cell); rightward-facing arrowhead points to a representative weakly-fluorescent, oval-shaped cell with a “fried egg” appearance (mast cell); upward-facing arrow points to a tiny, weakly-fluorescent round cell. Scale bar is 100 µm in all panels.

Transgenic HDC-Cre mice were bred with Rosa26-lox-STOP-lox-tdTomato reporter mice in order to generate offspring carrying the two transgenes (HDC/Tmt mice). The removal of the lox-flanked transcriptional STOP cassette by Cre recombinase results in the expression of the fluorescent Tmt protein specifically in cells expressing Cre recombinase. As Cre recombinase expression is directed by HDC transcriptional regulatory elements, Tmt should report on the location of histamine-producing cells. Fluorescence microscopy was utilized to verify the expected specificity of Cre recombinase activity within histaminergic cells of HDC/Tmt mouse stomachs. Three distinct cell types with Tmt fluorescence were observed in the stomach (Figure 1). Most labeled cells within the gastric mucosa were intensely fluorescent and displayed a mostly elongated (not-rounded) morphology, which is consistent with the known shape of ECL cells (Figure 1, leftward-facing arrow) [7], [43]. These intensely fluorescent, elongated cells were localized mainly to the glandular bases, with a fewer number of additional cells scattered within the glandular necks and apices. Such was consistent with previously published descriptions of enterochromaffin-like (ECL) cell distribution [7]. Within the gastric submucosa, muscularis, and less often, the mucosa, a generally weaker fluorescence was also observed within an oval-shaped cell type that had the appearance of a “fried egg”, with a dim fluorescent cytoplasm surrounding a more intensely fluorescent nucleus, as is commonly observed for mast cells (Figure 1, rightward facing arrowhead) [40]. The Tmt fluorescence was much brighter for the elongated, ECL cells (resulting in a yellowish, over-exposed center) than for the oval-shaped mast cells. In the submucosa, muscularis, and occasionally also the mucosa, there was a third population of weakly fluorescent cells with a tiny round shape, smaller than the mast cells (Figure 1, upward-facing arrow).

**Figure 2:**
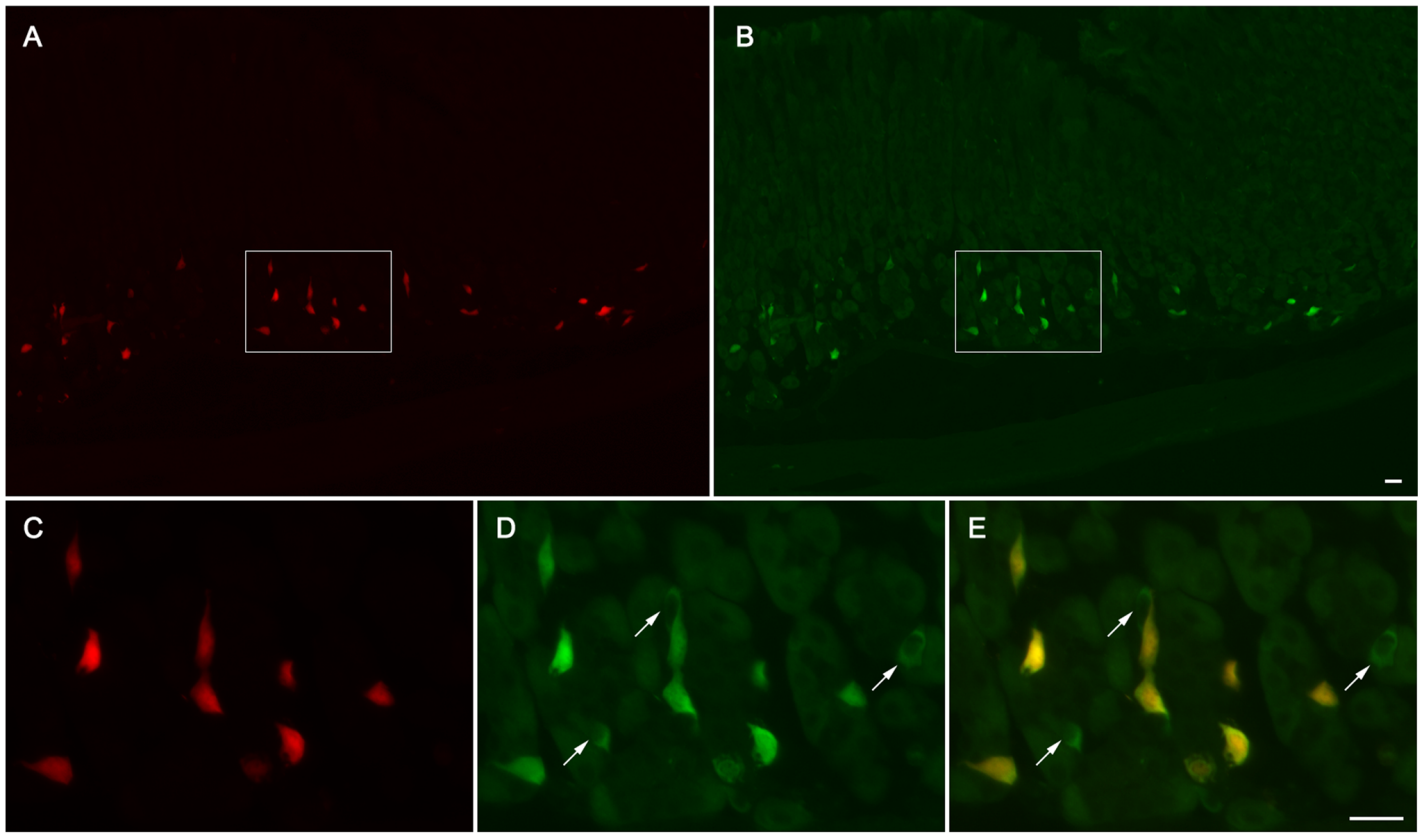
Co-localization of Tmt and HDC within the gastric oxyntic mucosa of HDC/Tmt mice. A–B) Low magnification photomicrographs showing the expression of Tmt fluorescence (A) within HDC-immunoreactive cells (B); the optics used to take these photomicrographs were such that only the elongated cells are visualized, and not the oval/fried egg-shaped cells or tiny round cells. C–E) High magnification views demonstrate that most (or in this region, all) cells with HDC-immunoreactivity (D) also express Tmt fluorescence (C). Arrows in (D) and in the overlay (E) indicate representative HDC-immunoreactive cells that lack Tmt fluorescence. Scale bar in (B) is 20 µm and also applies to (A). Scale bar in (E) is 20 µm and also applies to panels (C) and (D).

In order to confirm an HDC-specific expression of Cre recombinase activity, co-localization of HDC-immunoreactivity with Tmt fluorescence (as a reporter for Cre recombinase activity) was performed on stomach sections from HDC/Tmt mice (Figure 2, Table 1). Co-localization of HDC-immunoreactivity with Tmt fluorescence in gastric mucosal cells was quantified, revealing that nearly all (94.5% of) cells with Tmt fluorescence were also immunoreactive for HDC, while 73.4% of cells immunoreactive for HDC displayed Tmt fluorescence (Table 1). All three Tmt-fluorescent cell types were included in the co-localization analysis. Of note, Figure 2, depicts co-localization within the elongated ECL cells alone; for these photomicrographs, the optics were chosen to clearly distinguish the outlines of the ECL cells, and as such, the intensity of the fluorescence was lowered (as compared to that used in Figure 1), such that the more weakly-fluorescent, oval/fried egg-shaped mast cells and the tiny, weakly-fluorescent round cells were no longer visualized. None of the tiny round cells contained HDC-immunoreactivity, and as yet, the identity of these cells is unclear.

**Table 1:** Co-expression of Tmt and HDC within the gastric body mucosa of HDC/Tmt mice.

	% of cells with Tmt fluorescence co-expressing HDC-immunoreactivity	% of HDC immunoreactive cells co-expressing Tmt fluorescence
gastric mucosa	94.5±1.4	73.4±2.0

The data are reported as the mean percentage ± SEM for the gastric mucosa in the stomach at 4 different planes with 5 fields counted per plane; each plane is separated by at least 70 µm (n = 5 mice).

**Figure 3:**
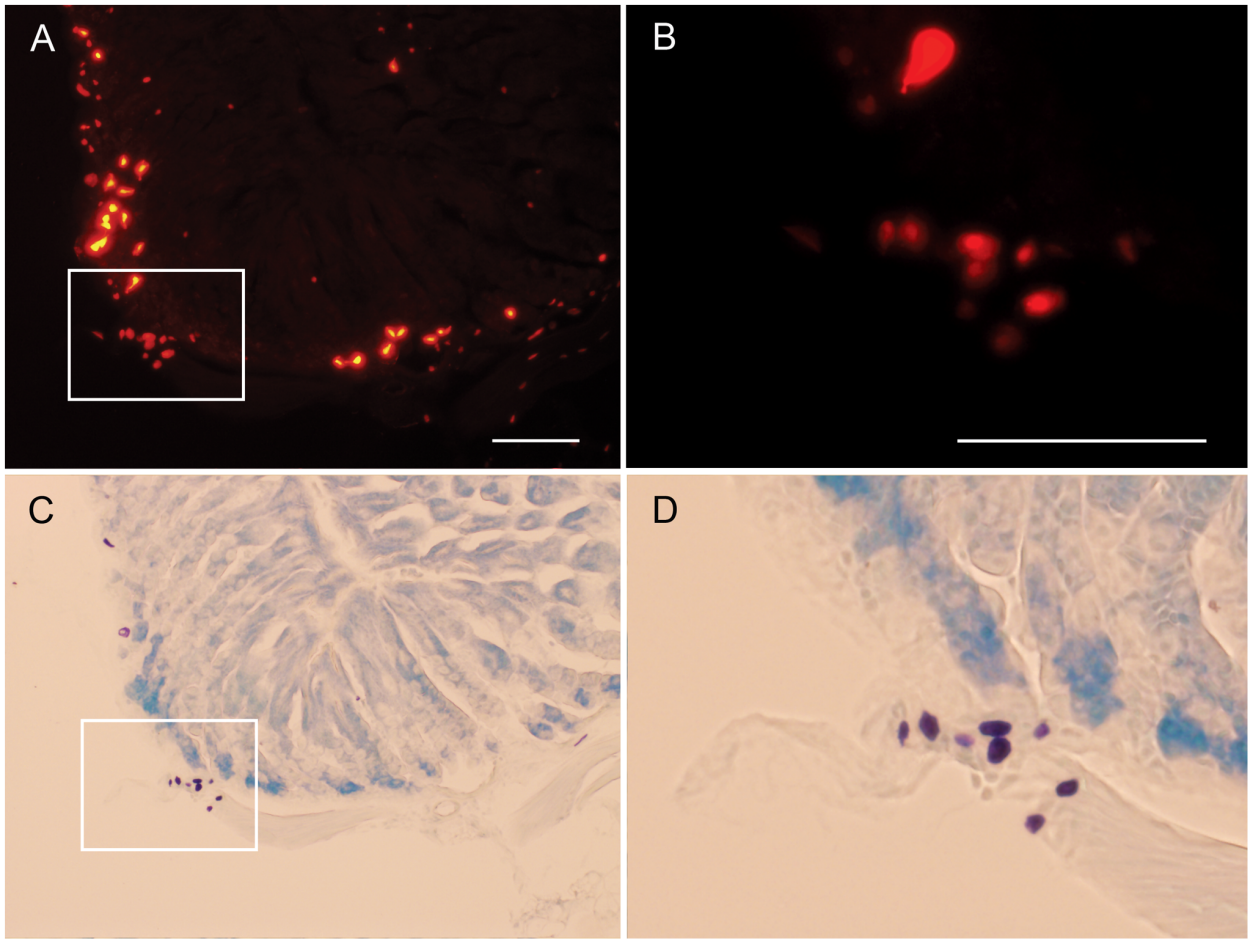
Expression of Tmt within mast cells of the stomach. A–B) Expression of Tmt fluorescence within the HDC/Tmt mouse stomach. C–D) Exclusive expression of toluidine blue stain within those HDC-immunoreactive cells with an oval/fried egg shape. Scale bar in (A) is 100 µm and also applies to (C). Scale bar in (B) is 100 µm and also applies to (D).

With the aim of further verifying that mast cells in the stomach display Tmt fluorescence, toluidine blue staining was performed on stomach sections of HDC/Tmt mice (Figure 3). Toluidine blue binds to heparin in the cytoplasmic granules of mast cells, thus distinctly labeling them a blue or purple color. Toluidine blue staining was observed in nearly all the Tmt fluorescent cells with the oval/fried egg-shaped appearance (Figure 3). Toluidine blue did not stain the more intensely fluorescent, elongated ECL cells of the mucosa, nor did it stain the weakly-fluorescent tiny round cells within the mucosa, submucosa and muscularis.

### Quantitative PCR on FACS-separated gastric mucosal histaminergic cells

**Figure 4:**
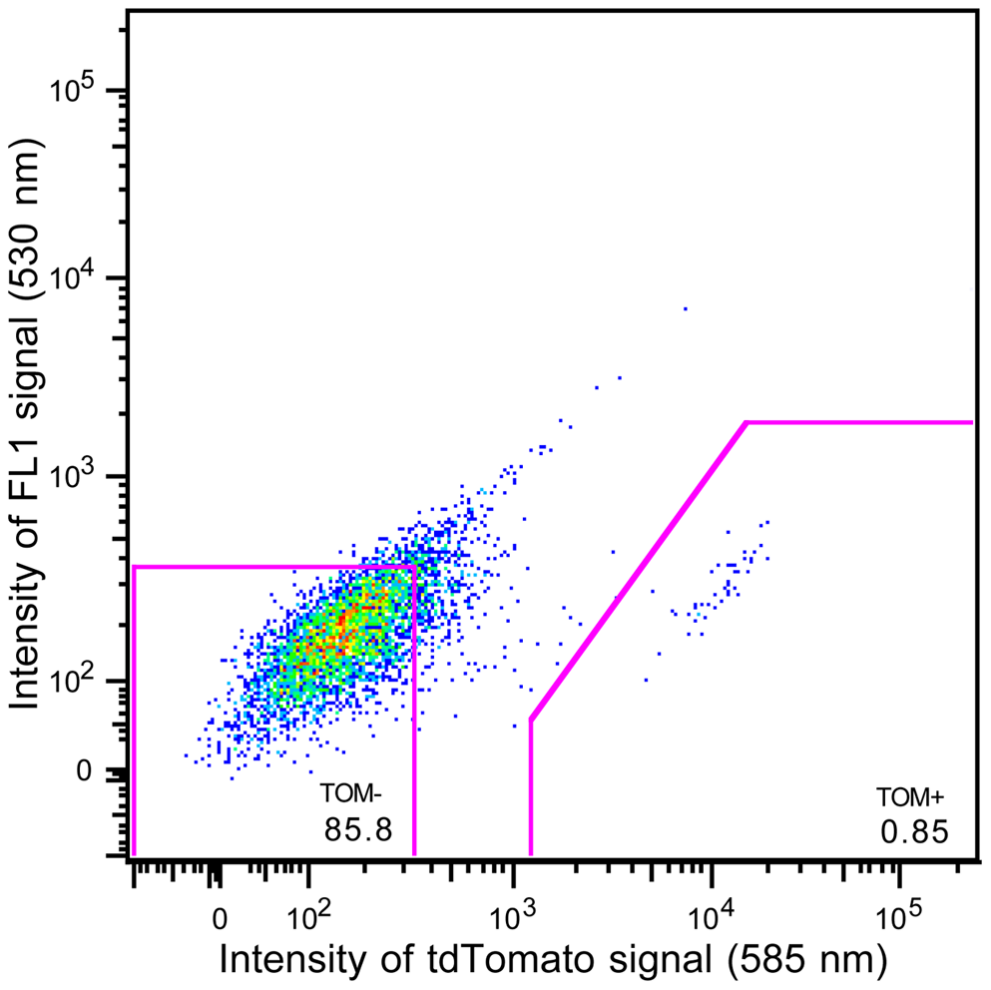
Fluorescence activated cell sorting of gastric mucosal cells. Graphical representation of FACS of mucosal cells from one representative set of HDC/Tmt mice, indicating cells collected as part of the Tmt-enriched pool (0.85% of the total number of sorted, living cells) and those collected as part of the Tmt-negative pool (85.8% of the total number of sorted, living cells).

To further characterize the gastric mucosal histaminergic cells, we again took advantage of the Tmt fluorescence signal present in the HDC/Tmt mice, this time as a means of isolating an enriched population of these cells. Cells comprising the gastric mucosa of HDC/Tmt mice were first enzymatically and mechanically dispersed and subsequently were submitted for FACS analysis to generate a population of enriched histaminergic cells. This was repeated for a total of 10 separate preparations of gastric mucosal cells. Cells displaying the highest intensity fluorescence were collected as “Tmt-enriched” pools, while cells with the lowest intensity fluorescence were collected as “Tmt-negative” pools (Figure 4). Next, mRNA was isolated from these FACS-separated populations, and the expression levels of various gastric endocrine cell, gastric non-endocrine cell, and mast cell markers were determined using quantitative RT-PCR; mRNAs of interest also were determined in samples of non-sorted, whole mucosa preparations (Table 2).

**Table 2:** Relative mRNA expression levels of various mRNAs in gastric mucosa and FACS-separated pools of gastric mucosal cell populations.

	Mucosa	Tmt-enriched pools	Tmt-negative pools
tomato (Tmt)	*undetermined* #	61.74±14.09***	*1.0±0.23*
			*(30.82±0.23)*
histidine decarboxylase	1.0±0.38	25.44±2.15***	0.08±0.03
(HDC)	(23.91±0.35)		
PAC1	1.0±0.28	3.91±1.07*	0.14±0.06
	(23.81±0.33)		
tryptase beta-2 (Tpsb2)	1.0±0.62	619.71±239.22*	4.06±2.81
	(24.15±.80)		
chromagranin A	1.0±0.37	5.57±1.45*	0.63±0.42
	(18.42±0.33)		
prohormone convertase 1/3	1.0±0.43	5.85±1.89*	1.37±0.49
	(22.73±0.36)		
prohormone convertase 2	1.0±0.20	3.13±0.79*	0.90±0.13
	(22.73±0.17)		
calpain 8	1.0±0.11	0.18±0.14	0.48±0.07
	(20.05±0.12)		
H+/K+ ATPase β-subunit	1.0±0.13	0.25±0.13	0.90±0.34
	(16.18±0.28)		
GIF	1.0±0.27	0.27±0.08	1.34±0.28
	(17.59±0.29)		
pepsinogen F	1.0±0.12	0.59±0.32	0.80±0.19
	(20.96±0.18)		
ghrelin	1.0±0.21	0.29±0.06	0.90±0.14
	(15.95±0.20)		
somatostatin	1.0±0.30	0.33±0.18	0.59±0.26
	(19.24±0.32)		
tryptophan hydroxylase	1.0±0.63	649.16±172.47***	18.76±1.20
	(24.44±1.0)		
gastrin	1.0±0.42	643.25±304.92*	1.11±0.20
	(21.11±0.62)		

All values are normalized to the housekeeping gene cyclophilin. With the exception of Tmt, each value represents the amount of mRNA relative to that within gastric mucosa preparations from C57BL6/J mice.

# The mRNA level was lower than the detection limit; because Tmt is not found in (wild-type) C57BL6/J mice, the value of Tmt in the Tmt-enriched pools represents the amount of mRNA relative to that of Tmt-negative pools.

* P<0.05, ***P<0.001 as compared to mucosa (or in the case of Tmt, as compared to Tmt-negative pools) when determined by Dunnett's post-hoc analysis.

Values in parenthesis denote the mean ± SEM of threshold cycles. (n = 3–4 FACS preparations/gene).

As expected, Tmt mRNA was found to be highly expressed within the Tmt-enriched pools as compared to that within Tmt-negative pools, suggesting a successful FACS enrichment of our target cells. High levels of HDC expression were observed in the Tmt-enriched populations, verifying that the cells marked with Tmt indeed express significant levels of HDC. Significant elevations in PACAP type 1 receptor (PAC1) a marker for ECL cells, and tryptase beta 2 (Tpsb2), a marker for mast cells, were observed in Tmt-enriched populations [44], [45], [46]. Common markers of endocrine lineage such as chromogranin A, prohormone convertase 1/3, and prohormone convertase 2 also were present at elevated levels in Tmt-enriched populations. As expected, there was no significant enrichment in the expression of markers for common non-endocrine gastric mucosal cell types such as calpain-8 (a marker of mucus-secreting pit cells), H^+^/K^+^ ATPase β-subunit (a marker of parietal cells), or gastric intrinsic factor and pepsinogen F (markers of zymogenic and parietal cells) [47], [48], [49]. There also was no significant enrichment in the expression of markers for two separate gastric endocrine cells types, including ghrelin (a marker for X/A-like, ghrelin cells) and somatostatin (a marker for D cells) [50], [51].

Interestingly, within the Tmt-enriched pools, a high amount of tryptophan hydroxylase 1 mRNA was detected. Tryptophan hydroxylase 1 catalyzes the rate-limiting step in the synthesis of serotonin, and thus is commonly used to distinguish serotonergic EC cells from other gastric mucosal endocrine cell types. That said, tryptophan hydroxylase 1 also is found in mast cells, which are an expected component of the Tmt-enriched pools and which produce not only histamine, but also serotonin [52], [53].

**Figure 5:**
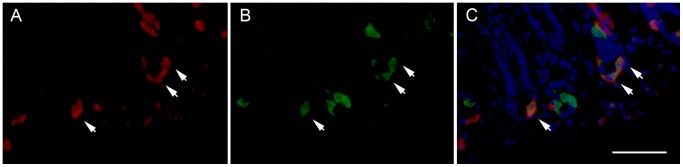
Co-localization of HDC with gastrin by dual-label immunofluorescence within the distal gastric mucosa. A few HDC-immunoreactive cells (B) co-express gastrin-immunoreactivity (A), and vice versa, as indicated by arrowheads as well as the yellow-orange color in the overlay (C). Blue color represents DAPI nuclear stain (C). Scale bar is 50 µm and applies to all panels.

Also noted within Tmt-enriched pools was elevated expression of gastrin mRNA, which is a marker for yet another gastric mucosal endocrine cell type, the G-cell. Gastrin and HDC co-localization has been previously described in the rat stomach [54], although to our knowledge, not in mice. In order to verify that HDC and gastrin co-localization is a usual occurrence in mice that are not genetically manipulated, dual-label immunohistochemistry on stomachs from C57Bl6/J mice was performed for HDC and gastrin. Indeed, within the gastric antrum (the distal part of the stomach), a small number of HDC-immunoreactive cells also demonstrated gastrin-immunoreactivity, and vice versa, supporting the existence of a subset of histaminergic gastric cells that co-express gastrin (Figure 5; Table 3).

**Table 3:** Co-expression of HDC and gastrin within the gastric antrum mucosa of mice.

	% of cells with HDC-immunoreactivity co-expressing gastrin-immunoreactivity	% of cells with gastrin-immunoreactivity co-expressing HDC-immunoreactivity
gastric antrum	29.1±2.1	17.3±1.7

The data are reported as the mean percentage ± SEM for the gastric antrum in the stomach at 8 different planes with 5 fields counted per plane; each plane is separated by at least 70 µm (n = 3 mice).

### Characterization of HDC-expressing cells in the histaminergic regions of the brain

**Figure 6:**
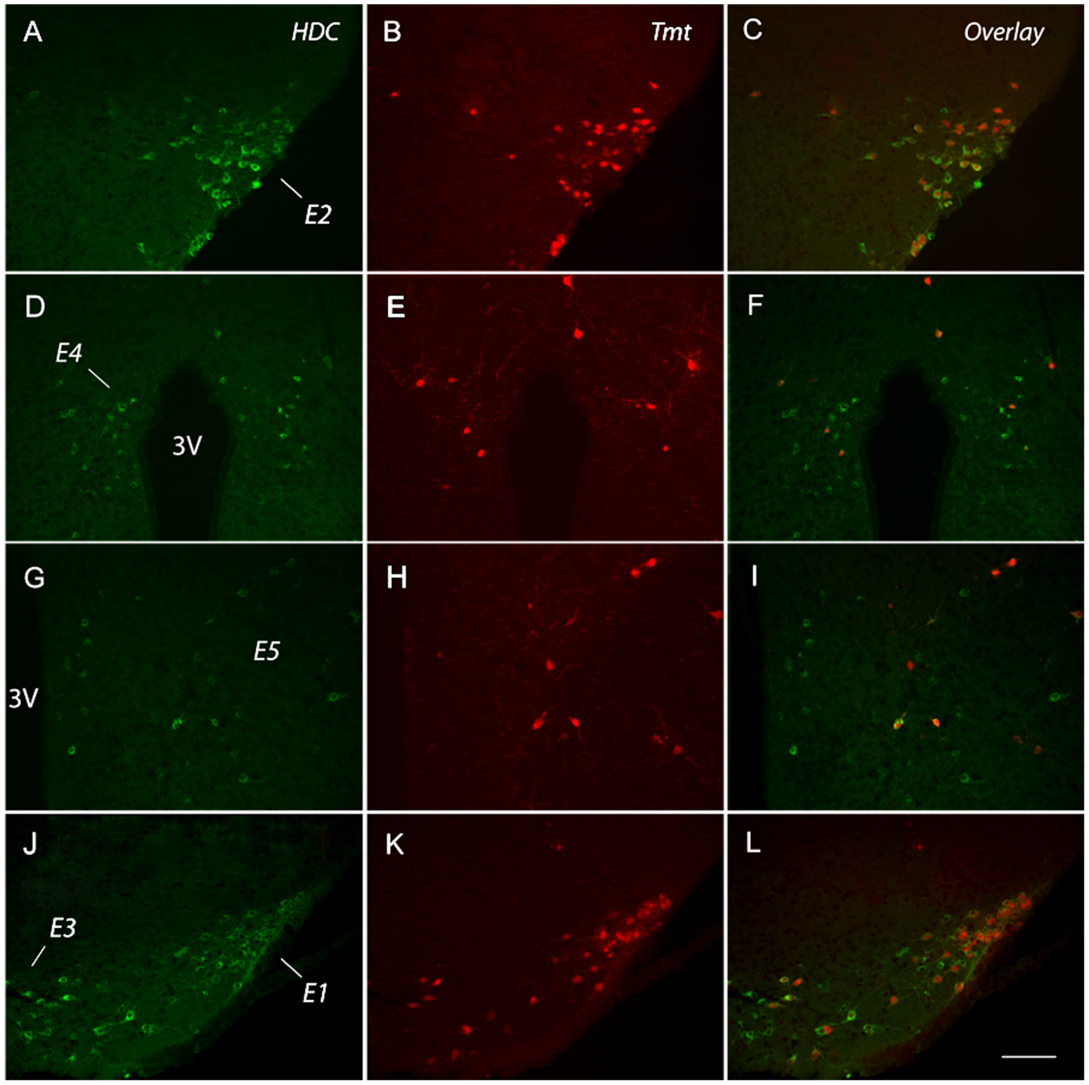
Expression of Tmt and HDC within the tuberomamillary nucleus histaminergic regions E1–E5 in HDC/Tmt mice. A,D,G,J) HDC-immunoreactive cells of representative sections for each histaminergic region. B,E,H,K) Tmt fluorescence exhibited in the same representative sections as shown in the left column. C,F,I,L) Overlay of the two images representing the co-localization of HDC immunoreactivity and Tmt fluorescence. Scale bar is 50 µm and applies to all panels. (*3V – 3^rd^ ventricle; in each column, sections arranged from the top to bottom as more rostral to more caudal)*.

Along with the gastric mucosa of the stomach, co-localization of HDC-immunoreactivity and our reporter of Cre recombinase activity (Tmt fluorescence) in HDC/Tmt mice was quantified in the brain (Figure 6 and Table 4). Percentages of co-localization were determined for each of the five distinct histaminergic regions within the tuberomamillary nucleus of the brain: regions E1–E5 [35]. Of note, previously, we reported the expression of Cre recombinase activity in the brain of HDC/Tmt mice, although this determination was restricted to the ventrolateral tuberomamillary nucleus (E2 region) alone. The caudal E3 group represented the tuberomamillary nucleus region with the highest percentage of HDC-immunoreactive cells also exhibiting Tmt fluorescence (82.1%; Table 4). Within the lateral histaminergic regions, E1 and E2, 80.8% and 81.8% of HDC-immunoreactive cells also exhibited Tmt fluorescence, respectively. The more medial histaminergic regions E4 and E5 displayed lower levels of co-localization, with 54.9% and 43.2% of HDC-immunoreactive cells also showing Tmt-fluorescence, respectively. Nearly all of cells with Tmt fluorescence within regions E1–E5 displayed HDC immunoreactivity, with % co-localization ranging from 90.6% to 97.5%.

**Table 4:** Co-expression of Tmt and HDC within the tuberomamillary nucleus of HDC/Tmt mice.

Histaminergic Brain Region	% of cells with Tmt fluorescence co-expressing HDC-immunoreactivity	% of HDC immunoreactive cells co-expressing Tmt fluorescence
E1	91.6±3.6	80.8±3.8
E2	97.5±1.0	81.8±4.0
E3	90.6±4.2	82.1±4.2
E4	92.3±2.7	54.9±5.7
E5	91.1±3.7	43.2±3.3

The data are reported as the mean percentage ± SEM for histaminergic brain regions E1–E5, each section separated by 80 µm; every section containing histaminergic regions in the hypothalamus was counted (n = 3 mice).

**Figure 7:**
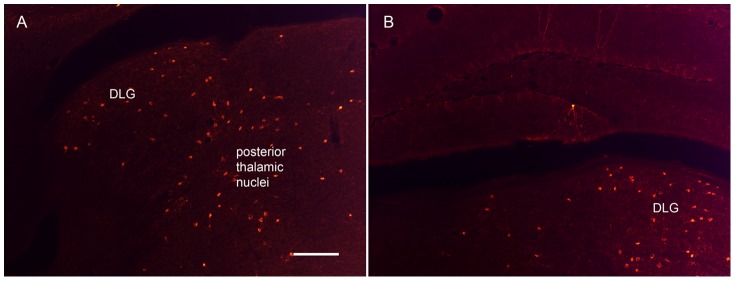
Non-specific expression of Tmt in the brain. A–B) Photomicrographs depicting presumed non-specific Tmt fluorescence (in cells without HDC-immunoreactivity) in the dorsal lateral geniculate nucleus (DLG), posterior thalamic nuclei, and hippocampus. Scale bar is100 µm and applies to both panels.

Of note, when viewing sections throughout the whole brain, Tmt expression was observed in several cells localized to the dorsal lateral geniculate nucleus (DLG) of the thalamus and the posterior thalamic nuclei, as well as rare hippocampal cells (Figure 7). These regions are not known to, nor did they in the current study, express HDC-immunoreactivity, suggesting likely non-specific Cre recombinase expression in these regions.

## Discussion

Histamine serves as an important signaling molecule for a group of neurons localized to the tuberomamillary nucleus of the brain, for ECL endocrine cells populating the gastric mucosa, and for mast cells and the related basophils which form an important part of the immune system. As mentioned, these histaminergic systems play key roles in many different processes, including sleep, digestion and host immune defense. The sparsely distributed expression of these different populations has undoubtedly hampered a more rapid exploration of the normal physiology and pathophysiology of these systems. The HDC-Cre transgenic mouse model described here is capable of being a useful tool for studying these multiple histaminergic regions and systems. In the present study, we have used a combination of histochemistry and fluorescence activated cell sorting together with quantitative PCR to validate the appropriate expression of Cre recombinase activity within the histaminergic cells populating the brain and stomach. By breeding the HDC-Cre mice with td-Tomato reporter mice, we were able to easily visualize cells expressing Cre recombinase activity in the form of Tmt fluorescence. Within the stomachs of HDC/Tmt mice, those cells exhibiting the brightest Tmt fluorescence in the mucosal layer were mainly localized to the base of the glandular region and had an elongated morphology reminiscent of the distribution and shape of ECL cells [43]. We succeeded in directing Cre recombinase expression to the vast majority of gastric histaminergic cells, as 73.4% of mucosal cells containing HDC-immunoreactivity also exhibited Tmt fluorescence. Taking advantage of the fluorescent labeling of the histaminergic cells in the gastric mucosa, highly enriched populations of histaminergic cells were isolated using FACS, of which subsequent qPCR analyses confirmed the presence of known markers for both ECL cells and mast cells. Gastric mast cell expression of Cre recombinase activity was confirmed by co-localization of the tdTomato signal with toluidine blue staining, which is a known marker of mast cells. We also demonstrated expression of Cre recombinase activity within all the various regions of the tuberomamillary nucleus of the brain. Greater than 80% of the histaminergic neurons of the E1, E2 and E3 regions of the tuberomamillary nucleus contained Cre recombinase activity while less of the histaminergic neurons of the E4 and E5 regions (55% and 43%, respectively) co-localized with Cre recombinase activity. Presumed ectopic Cre recombinase expression that was not co-localized with HDC-immunoreactivity was observed within a small region of the thalamus, rare hippocampal neurons and an otherwise un-identified population of tiny, round cells within the gastric submucosa, gastric muscularis layer, and occasionally, the gastric mucosa. It is possible, although we believe unlikely, that these non-specific populations of fluorescent cells result from embryonic HDC activity that is no longer present in adult cells. This is especially true for the tiny, round gastric cells, as HDC expression has been found to be undetectable in the stomach during the embryonic stages of development [35]. Additionally, although the tiny, round cells are likely to have been separated into the Tmt-enriched pools of mucosal cells, the histochemical analysis depicting only rare occurrences of these cells within the mucosa suggests that they are not contributing substantively to the presented quantitative PCR analyses.

One curious observation revealed during the validation of the HDC-Cre mouse model was the finding of relatively high gastrin expression in the Tmt-enriched population of isolated gastric mucosal cells. Although gastrin and HDC co-localization had previously been reported in the rat stomach, to our knowledge, this finding has not been replicated in the mouse or elaborated upon since the initial discovery [54]. It might be interesting for future studies to determine if these histamine- and gastrin-co-expressing cells have an electron microscopic appearance more similar to ECL cells or more similar to G-cells as well as to determine if they serve functions distinct from those ECL cells or G cells that express either histamine or gastrin alone.

The utility of the now validated HDC-Cre transgenic mouse model is potentially broad. As mentioned, Cre-mediated expression of Tmt fluorescence has already permitted ventrolateral tuberomamillary nucleus histaminergic cells to be studied electrophysiologically [37]. Now that expression of Cre recombinase within histaminergic neurons also has been confirmed in the other regions of the tuberomamillary nucleus, electrophysiological properties of those neuronal subpopulations also can be examined. The concept of functionally divergent populations as opposed to one cohesive population of histaminergic neurons creates exciting possibilities for a new understanding of histaminergic signaling in the central nervous system. Combining the selective expression of Cre recombinase activity with electrophysiology as well as histochemistry and other biochemical techniques can also be taken advantage of to help further clarify the neuroanatomical circuits, physiological processes and behaviors in which these various tuberomamillary regional populations participate. Similarly, within the stomach, the capability to easily detect ECL cells and mast cells using this new mouse tool opens up the possibilities for novel, yet practical studies. The validation of an HDC reporter mouse that accurately reflects histaminergic cells in the stomach will allow researchers in the field to feasibly pursue studies of ECL function, morphology, distribution, gene expression in response to an endless number of stimuli or treatments.
